# Behavioral, Ecological and Genetic Differentiation in an Open Environment—A Study of a Mysid Population in the Baltic Sea

**DOI:** 10.1371/journal.pone.0057210

**Published:** 2013-03-01

**Authors:** Martin Ogonowski, Jon Duberg, Sture Hansson, Elena Gorokhova

**Affiliations:** 1 Department of Systems Ecology, Stockholm University, Stockholm, Sweden; 2 Department of Applied Environmental Science, Stockholm University, Stockholm, Sweden; Technical University of Denmark, Denmark

## Abstract

Diel vertical migration (DVM) is often assumed to encompass an entire population. However, bimodal nighttime vertical distributions have been observed in various taxa. Mysid shrimp populations also display this pattern with one group concentrated in the pelagia and the other near the bottom. This may indicate alternative migratory strategies, resembling the seasonal partial migrations seen in birds, fishes and amphibians, where only a subset of the population migrates. To assess the persistence of these alternative strategies, we analyzed the nitrogen and carbon stable isotope signatures (as proxies for diet), biochemical indices (as proxies for growth condition), and genetic population divergence in the Baltic mysid *Mysis salemaai* collected at night in the pelagia and close to the bottom. Stable isotope signatures were significantly different between migrants (pelagic samples) and residents (benthic samples), indicating persistent diet differences, with pelagic mysids having a more uniform and carnivorous diet. Sequencing of the mitochondrial cytochrome subunit I (COI) gene showed genetic differentiation attributable to geographic location but not between benthic and pelagic groups. Divergent migration strategies were however supported by significantly lower gene flow between benthic populations indicating that these groups have a lower predisposition for horizontal migrations compared to pelagic ones. Different migration strategies did not convey measurable growth benefits as pelagic and benthic mysids had similar growth condition indices. Thus, the combination of ecological, biochemical and genetic markers indicate that this partial migration may be a plastic behavioral trait that yields equal growth benefits.

## Introduction

In aquatic ecosystems, processes that mediate energy flux between benthic and pelagic environments are crucial in maintaining ecosystem integrity and seasonal productivity [1], [2]. An important mechanism of these fluxes is the diel vertical migration (DVM) performed by many freshwater and marine invertebrate species [3]. The movement pattern is usually manifested by an ascent to the upper part of the water column at dusk and a descent to the deeper and darker waters at dawn. The main benefit of this behavior is that it enables feeding at a time and space when predation risk is low, which is strongly supported by the fact that many invertebrates avoid waters containing kairomones (fish scent) from predatory fish [4], [5], [6]. The trade-offs however, are often associated with a reduction in growth and reproduction as a consequence of lower temperatures and food availability in deeper and darker waters [7], [8]. The extent of DVM and consequently also the contribution to the benthic-pelagic coupling, however, varies widely among species.

Mysid shrimps (Crustacea, Mysidacea) are common in both marine and freshwater systems. They have strongly pronounced DVM and are capable of migrating several hundreds of meters from the bottom towards the surface at night [9]–[11]. Moreover, because they are opportunistic omnivores, they are able to feed in both benthic habitat (on detritus, benthic animals and algae; [12], [13], [14] and in the pelagia [14], [15]), although the food available in the pelagic zone generally is assumed to be of higher quality due to its higher nutrient content (e.g. [16], [17]).

In the Baltic Sea, there are three species of pelagic mysids: *Mysis mixta* Liljeborg, *Mysis relicta* Lovén and *Mysis salemaai* Audzijonyte and Väinölä [18]; all of them performing nocturnal diel vertical migrations. In the northern Baltic proper, *M. relicta* and *M. salemaai* are sympatric [18], [19]. Due to their morphological similarity, *M. salemaai* was until recently unknown and grouped with *M. relicta*, as were two other cryptic species from North America (*Mysis diluviana* and *Mysis segerstralei*; Audzijonyte and Väinolä 2005). This implies that earlier reports on *M. relicta* may in fact refer to a mixture of sibling species, in all areas where the cryptic species co-occur.

Even though DVM is a prominent feature of mysid behavior, a bimodal night-time vertical distribution in mysid populations has been observed (North American lakes: [20], [21]; the Baltic Sea: [13]) with part of the population staying close to the bottom, indicating that some individuals may be non-migrating. Morgan [20] assumed this group to consist mostly of recently moulted gravid females, although this was later rejected by Bowers [21] who observed that the demographic structure of the benthic part of the population was representative of the entire population. Another possible explanation for such a bimodal distribution with homogenous demographic structure is the existence of consistent intraspecific differences in resource and habitat use.

Intraspecific niche differentiation expressed as either morphological, behavioral, ontogenetic, genetic differentiation or some combination of the above [22], [23], is a strong diversifying force in nature and possibly even an important precursor to speciation (e.g. [22], [24], [25]) and has been shown to be correlated with the relaxation of either interspecific [25] or intraspecific competition [26], [27] and the existence of open niches or underutilized resources. Intraspecific differences in resource use are widespread across a variety of taxa [22], [23], being especially prominent in fish that commonly divide into benthic and pelagic feeders [24].

Although populations may inhabit varying discrete niches more or less permanently to specialize on particular food items (often with morphological adaptations) and thus decrease intraspecific competition [26], [27], seasonal or temporary niche shifts may be adopted by a subset of a population by migration to other habitats. This phenomenon is commonly referred to as partial migration; being described in an array of animal taxa (birds: [28]; mammals: [29]; amphibians: [30]; fish: [31]) where both strategies may yield equal fitness pay-offs [28] and be evolutionary stable [32]. However, the fundamental reasons for the adoption of a resident/migratory strategy seem to differ across species and systems. By ‘choosing’ to migrate, intraspecific competition may be temporarily relieved by an extension of the realized niche; i.e. a shift to more profitable or abundant prey instead of specialization on a typical resource [33] or it can be a way to decrease predation pressure [34]. The costs of migration can be associated with increased predation, energetic demands or a combination of both [33],[34]. Additionally, birds usually display some level of genetic linkage to migratory behavior [28], [35], whereas in fish, genetic coupling seems to be weak [36], [37] and the trait is suggested to be plastic, triggered mainly by frequency-dependent processes [34], [37], [38]. Size, growth rate and condition also seem to be correlated with the probability of adopting a migratory strategy [34], [37], [39]. Hence, ultimate and proximate causes of this behavior seem to be incoherent across animal groups and the understanding of partial migrations is still incomplete.

Although partial migrations, where animals adopt different life history strategies (migration or residency) have been described in seasonal environments, few studies have considered the possibility of such a migratory strategy in cases where migration is considered to be more or less regular, like DVM. Recently, Mehner and Kasprzak [40] proposed partial migration to be present in DVM performing fish, the authors did however not consider the persistence of habitat use by migrants/residents, making inferences on evolutionary and ecological consequences uncertain.

Here, we examine whether the observed bimodal night-time distribution in mysids can be explained by (i) interspecific differences in migratory behavior between the sibling species, (ii) an unsynchronized DVM within the homogenous population, i.e. non simultaneous migration towards the surface, or (iii) consistent, intraspecific divergent migratory tactics with distinct genetic morphs, thus resembling the partial migrations undertaken by many species of fish and birds. We address these hypotheses by examining the species composition of *Mysis* spp. in concert with their diet and growth condition in relation to their migratory behavior (pelagic vs. benthic) in a coastal area of the Baltic Sea. Once the absolute dominance of *M. salemaai* in both pelagic and benthic groups was established, we used mitochondrial DNA to assess the genetic structure of our sampled population. To assay feeding habitats of the mysids, we used a stable isotope approach, a useful tool in studying food web interactions and movements between isotopically distinct food webs [41]–[43]. Consumers tend to be enriched in the heavier nitrogen isotope (^15^N) compared to their diet and this can be used to estimate trophic position within a food web [44], [45]. The carbon isotope signature, however, changes little throughout the trophic chain, providing an indicator of the utilized food source. This approach is particularly useful for delineating pelagic and benthic food sources [44], [46], [47]. In order to investigate diet variability in pelagic vs. benthic mysids, we compared the correlations between δ^15^N and δ^13^C. To assess differences in mysid growth condition between the habitats and stations, we used the protein:DNA ratio in individual mysids; this ratio has been successfully used as a surrogate marker of changes in individual growth related to cell size dynamics and gross protein synthetic activity in different crustaceans, including mysids (*M. diluviana*) [48], [49]. Also, the C:N ratio was used as a proxy for lipid accumulation.

## Materials and Methods

### Study area

**Figure 1:**
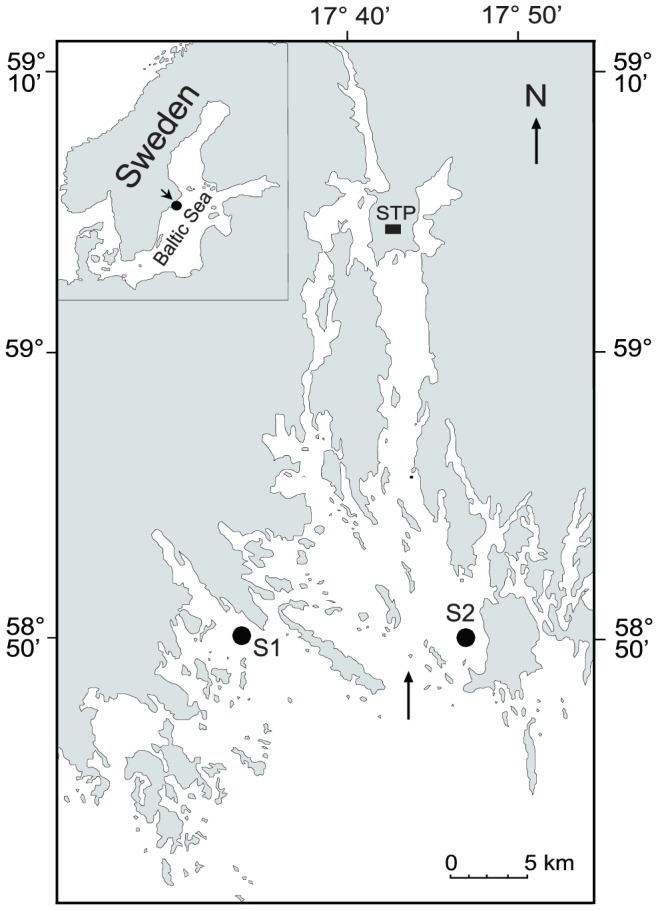
Map of study area. Map of the study area in the northern Baltic proper, showing sampling stations where mysids were collected in September 2008. STP = sewage treatment plant. The arrow indicates the main strait providing a connection between the areas in which stations S1 (58° 49′N, 17° 33′E) and S2 (58° 49′N, 17° 46′E) are located.

Mysids were sampled at two coastal stations (S1: 58° 49′N, 17° 33′E and S2: 58° 49′N, 17° 46′E; Fig.1), located in a coastal area of the northern Baltic proper. The stations are 30–35 m deep with stable surface salinity of 6–7 on the practical salinity unit scale. Station S2 is situated in the mouth of Himmerfjärden Bay influenced by nutrient discharge from a sewage treatment plant. The discharge is enriched in ^15^N, resulting in elevated concentrations of this isotope in producers and consumers of the food web, whereas Station S1 is not measurably affected by discharges from the sewage treatment plant [41], [50].

### Sampling and preservation

Samples were collected at night (22:00 to 04:00), 8-9-Sept-2008. No daytime samples were taken, because *Mysis* spp. stay very close to the bottom during the day [13], [51]. Pelagic mysids were collected with a Tucker trawl (effective opening = 0.25 m^2^) at a depth of 25±2 m. Benthic mysids, occurring 5–30 cm above the sediment surface, were collected with an epibenthic sled [52]. Both pelagic and epibenthic tows were taken along approximately 1 km long transects. Because mysids could not be identified to species in the field, approximately 60 mysids within the same size range (12.4–15.6 mm, representative of the population mean; mysids have a one year life-cycle in this part of the Baltic Sea [53]) were selected from the total collection at each station and habitat, rinsed in distilled water and frozen individually in Eppendorf™ tubes immediately after collection. The remaining mysids were stored in ∼60% ethanol (final concentration). A second subsampling was performed in the lab, where *Mysis relicta/salemaai* were separated from other mysids based on the morphological characteristics described by Mauchline [11]. Only frozen samples were selected for stable isotope analysis since alcohol preservation may affect isotopic ratios [54], whereas pleopods from both frozen and similar sized ethanol-preserved individuals were used for the genetic analysis.

### Species identification

*Mysis relicta* and *M. salemaai* were identified by amplification and sequencing of a 634 bp fragment of the mitochondrial cytochrome c oxidase subunit I (COI) gene. [18]. Altogether, 171 samples were analyzed, including all individuals used for SIA, 168 of them were amenable to amplification. To extract DNA, 100 µl of 6% Chelex 100 (BioRad*®*) was added to homogenized pleopod samples and heated for two hours at 60°C followed by 105°C for 10 min [55]. Then the samples were centrifuged for 2 min at 14 000 rpm and the supernatant was collected and stored at 4°C. Amplifications were performed using the specific *Mysis* primers: myshatD1516, 5′-cactctattttgtttttggrgcttg- 3′and myshatR2164, 5′-cttctgccaccttctttagctc- 3′ [18] using an MJ Research MiniCycler. In each PCR reaction, 46 µl of PCR master mix (Qiagen), 1 µl (10 µM) of each primer (Invitrogen) and 3 µl of template DNA were used. An initial denaturation was initiated for 3 min at 94°C, and then 30 cycles were run, each consisting of 45 s of denaturation at 94°C, 45 s of annealing at 55°C, and 1 min of extension at 72°C, followed by a final extension for 1 min at 72°C. A random selection of PCR products were visualized by electrophoresis in 1% agarose gel and stained with ethidium bromide in Tris-borate-EDTA buffer. All PCR products were purified using either a QIAquick Spin PCR purification kit (Qiagen) or Zymo-spin IC (Nordic biolabs). The resulting PCR product was sequenced on an ABI 3730 and 3130XL PRISM*®* DNA Analyzer (KI-gene sequencing facility, Solna, Sweden) using both the forward and the reverse primers (24 samples), which resulted in identical sequences, or only the reverse primer (rest of the samples). To assess sequencing error, 15 individuals were run twice and were invariably assigned to the same haplotype.

The sequences were visually inspected, aligned and trimmed in BioEdit v. 7. 0. 5. 3, resulting in a 469 bp sequence of sufficient quality used for further analysis. These sequences were subjected to a BLAST search (http://www.ddbj.nig.ac.jp/search/blast-j.html) using the NCBI BLAST function optimized for highly similar sequences (megablast) for species identification based on the highest alignment score.

### Sample processing and preparation for stable isotope analysis (SIA)

All mysids were sexed according to the sexual characteristics described in Mauchline [11]. If present, males were distinguished by an elongation of the 4^th^ pleopod and females by the presence of brood plates or marsupium. In absence of sexual characteristics, mysids were classified as juveniles. Frozen mysids were measured from the tip of the rostrum to the end of the last abdominal segment (body length, BL) using a caliper (±0.02 mm).

For SIA, both muscle tissue and purified chitin were used. Muscle tissue has a relatively low turnover and reflects the diet over most of the growth season in subadult *Mysis* [56], whereas chitin gives an integrated signal over a shorter period of time (usually a few weeks, corresponding to the last intermoult period). In preparation for analysis, abdominal muscle tissue was dissected from each mysid and transferred into pre-weighted tin capsules. All measurements and dissections were performed under a dissecting microscope. To avoid contamination, all dissections were performed on sterile petri dishes and with instruments cleaned with distilled water between each sample preparation. After dissection, the remaining parts from each mysid were transferred into labeled Pyrex tubes and stored desiccated until chitin extraction. The procedure of extracting chitin and determining its purity was based on methods described by Tsao and Richards [57] and DeNiro and Epstein [44]. Whereas muscle tissue was analyzed for each individual, the chitin samples had to be prepared by pooling 4–6 individuals per replicate to achieve an optimal sample size, which is why additional mysids of similar size from the same station/habitat were used for the chitin samples. The samples were finally dried at 60°C for 48 h, dry weight was determined to the nearest microgram using a Sartorius M3P microbalance and thereafter stored desiccated until stable isotope analyses.

### Stable isotope analysis (SIA)

Samples were analyzed for their relative abundance of stable carbon and nitrogen isotopes as well as per cent carbon and nitrogen using a PDZ Europa ANCA-GSL elemental analyzer interfaced to a PDZ Europa 20-20 isotope ratio mass spectrometer (Sercon Ltd., Cheshire, UK). Isotopic analyses were performed at UC Davis Stable Isotope Facility, University of California, Davis. The results are expressed in δ as parts per thousand (‰) according to equation 1



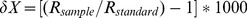
(1)


where X is ^15^N or ^13^C and R is the corresponding ratio ^15^N/^14^N or ^13^C/^12^C. The reference material used was atmospheric N_2_ and PeeDee Belemnite, respectively. Two working standards (homogenized mysid tissue) were run for every 42±2 samples (*n* = 28). The analytical precision for δ ^15^N and δ ^13^C were within the limits of±0.13‰ and±0.09‰ S. D. respectively. Analytical and method blanks were also included to control for possible analytical drift as well as contamination risk during laboratory work. No lipid correction was necessary as C:N ratio never exceeded 3.5 (Table 1), [58].

**Table 1:** Descriptive statistics of isotopic signatures, C:N and protein:DNA ratios.

Habitat	n		δ^15^N	δ^13^C	BL (mm)	C:N	Protein:DNA
Muscle							
S1-benthic	20	(15)	11.59±0.16	−18.96±0.22	14.26±0.86	3.28±0.02	39.1±17.1
S1-pelagic	17	(8)	11.71±0.18	−18.94±0.16	14.17±0.90	3.30±0.06	32.5±17.4
S1-total	37	(23)	11.64±0.18	−18.95±0.19	14.22±0.87	3.29±0.04	36.8±17.1
S2-benthic	17	(18)	12.42±0.32	−18.61±0.12	13.50±0.70	3.31±0.02	36.9±14.6
S2-pelagic	18	(16)	12.91±0.59	−18.37±0.20	14.31±0.81	3.29±0.02	43.9±20.9
S2-total	35	(34)	12.67±0.53	−18.49±0.20	13.92±0.86	3.29±0.02	40.2±17.9
Chitin							
S1-benthic	12		−8.72±0.26	−21.60±0.13	14.23±0.41		
S1-pelagic	5		−8.44±0.20	−21.71±0.17	14.22±0.54		
S1-total	17		−8.64±0.27	−21.63±0.15	14.23±0.43		
S2-benthic	3		−8.04±0.07	−20.92±0.07	13.51±0.12		
S2-pelagic	10		−7.81±0.39	−20.96±0.18	13.84±0.49		
S2-total	13		−7.86±0.35	−20.95±0.16	13.76±0.45		

Summary of isotopic signatures (δ^13^C, δ^15^N) for muscle tissue and chitin, mysid size (BL, mm), C:N ratios and protein:DNA ratios in *M. salemaai* collected in the pelagic and benthic environment at station S1 and S2. All data are presented as mean±standard deviation (SD). Numbers in parenthesis denote number of samples used for protein:DNA analysis.

### Protein:DNA ratio

Pleopods were dissected from ethanol-preserved mysids (8 to 18 per station/habitat group) and used to quantify total water soluble protein and DNA. The samples were homogenized in 130 µL of 1% N-laurylsarcosine using a FastPrep homogenizer with cooling function, and incubated for 2 h on a shaker. The protein and DNA concentrations (µg mL^−1^) were determined using microplate-based bicinchoninic acid assay (BCA, Pierce Ltd.) with bovine serum albumin as standard and fluorometric high-range RiboGreen (Molecular Probes, Inc., Eugene, OR) assay with calf thymus DNA as standard [59], respectively. All samples were analyzed in duplicates using a FLUOstar Optima microplate reader with absorbance and fluorescence configuration for protein and DNA, respectively, and protein:DNA ratio was calculated for each sample using averaged values.

### Statistical analyses

We performed a factorial multivariate analysis of variance/covariance (MANOVA/MANCOVA) to test for effects of body size and differences between the benthic and pelagic samples on a combination of carbon and nitrogen isotopic ratios. We then used a factorial analysis of variance/covariance (ANOVA/ANCOVA, via generalized least squares models (GLS) of the ‘nlme’ package in R [60]) to investigate each isotope ratio separately. In all cases, we started with a full model, including all possible interactions whereby we dropped insignificant terms to minimize the Akaike Information Criterion score (AIC), which is a measure of the goodness of fit of an estimated model. The most parsimonious models (lowest AIC) of muscle tissue isotopic signatures were expressed as a function of station (S1 or S2), habitat (pelagic and benthic zone), body length (BL) and a BL×station interaction. Models for chitin did not include BL or any interactions. To assess differences in protein:DNA ratio between stations and habitats, we performed an ANCOVA with protein concentration as the response variable, and DNA concentration and BL as covariates. The protein:DNA ratio was thus split into its components as this increases the sensitivity of the analysis [49] and the variables were log transformed to ensure linearity and homogeneity. Differences in C:N ratios were analyzed with an ANCOVA using BL as a covariate. C:N data were transformed by reciprocal square root transformation in order to achieve normality. Unequal variances between groups were checked in all analyses using Bartlett's tests and if found, accounted for by the use of Pillai's test statistic for the MANOVA/MANCOVA [61] or the addition of a variance structure to the ANOVA/ANCOVA models [62]. Diagnostic plots of normalized residuals vs. fitted values were performed to investigate any departures from model assumptions. All analyses were performed in R v. 2.13.0 R (R development core team 2011).

### Population genetic structure

We assessed global and pairwise genetic differentiation using Fisher's exact tests with 10 000 dememorisation steps and an analysis of molecular variance (AMOVA) based on genetic distances between haplotypes [63]. To discern if the genetic variation could be apportioned more to geographic location (stations) or habitat (benthic-pelagic), we performed two higher level AMOVA analyses. One with station as the grouping factor (AMOVA I) and the second with habitat as the grouping factor (AMOVA II). Estimates of the genetic differentiation index *F_ST_* [64] and corresponding p-values were calculated by 10 000 permutations; to correct for multiple testing, the modified FDR procedure [65] was applied. All analyses were performed in Arlequin v. 3.5.1.2 [66]. A minimum spanning tree (MST) based on the number of nucleotide differences between haplotypes was also constructed using a distance matrix from Arlequin in Hapstar v. 0.6 [67] to visualize the network of interrelationships between the haplotypes.

Given the relatively low *F_ST_* values observed, the statistical power of our analysis to reject the null hypothesis of genetic homogeneity was assessed by a power-test (POWSIM) [68] testing for a hypothetical true differentiation quantified as *F_ST_* = 0.01. We used the default settings in POWSIM, modeling over 10 generations (*t*) and with an effective population size (*N_e_*) of 500. The analysis showed that our dataset had high power (97.6%) of detecting global population structure even at a low level of genetic differentiation (*F_ST_* = 0.01).

## Results

### Species composition

The mysid community was heavily dominated by *M. salemaai* (95% of the total sequenced specimens; *n* = 168), with a few *M. relicta* (*n* = 8) that were all caught pelagically. Therefore, the analyses of stable isotope signatures and population genetic structure reported here are focused exclusively on *M. salemaai*.

### Isotopic composition

Mysids analyzed for stable isotopes all consisted of juveniles and immature males where the latter contributed 20–48% of the total sample size and were similar in size with a mean BL of 14.06±0.78 mm (±S.D.). However, individuals in the benthic group from station S2 were on average 0.8 mm smaller than individuals in the pelagic group (Table 1. Wilcoxon rank sum test: W_(1, 1)_ = 81, *p* = 0.017).

The combined isotopic signal in muscle tissue was significantly affected by mysid size where isotopic ratios increased with BL. This effect was however only present at station S2 (linear regression: *F*_1, 33_ = 28.2, *p*<0.0001, *r^2^* = 0.44 and *F*_1, 33_ = 23.9, *p*<0.0001, *r^2^* = 0.42; δ^15^N and δ^13^C respectively), which explains the significant interaction between station and BL (Table 2). Furthermore, as the benthic sample at station S2 was smaller compared to the pelagic, part of the variation explained between the S2 habitats was attributable to differences in mysid size.

**Table 2:** ANCOVA/ MANCOVA results.

Dependent	Predictor	Estimate	S.E	*t*	*Pillai's trace*	*p*
*Muscle*						
δ^15^N x δ^13^C	station				0.32	<0.0001
	habitat				0.79	<0.0001
	BL				0.21	0.0003
	station*BL				0.25	<0.0001
δ^15^N	station	−3.52	1.11	−3.16		0.0024
	habitat	0.14	0.05	2.55		0.0132
	BL	0.01	0.03	0.36		0.7228
	station*BL	0.33	0.08	4.04		0.0001
δ^13^C	station	−1.71	0.70	−2.44		0.0174
	habitat	0.08	0.04	1.73		0.0888
	BL	−0.02	0.03	−0.69		0.4940
	station*BL	0.16	0.05	3.11		0.0027
*Chitin*						
δ^15^N x δ^13^C	station				0.65	<0.0001
	habitat				0.85	<0.0001
						
δ^15^N	station	0.66	0.12	5.55		<0.0001
	habitat	0.25	0.12	2.11		0.0441
						
δ^13^C	station	0.72	0.06	11.3		<0.0001
	habitat	−0.08	0.06	−1.13		0.2010

Most parsimonious (lowest AIC) model results of stable isotopes (δ^15^N and δ^13^C) modeled jointly (MANCOVA) or separately (ANCOVA) as a function of station (S1, S2), habitat (benthic, pelagic) and interactions therein for muscle tissue and chitin. Sums of squares are calculated using type III SS.

**Figure 2:**
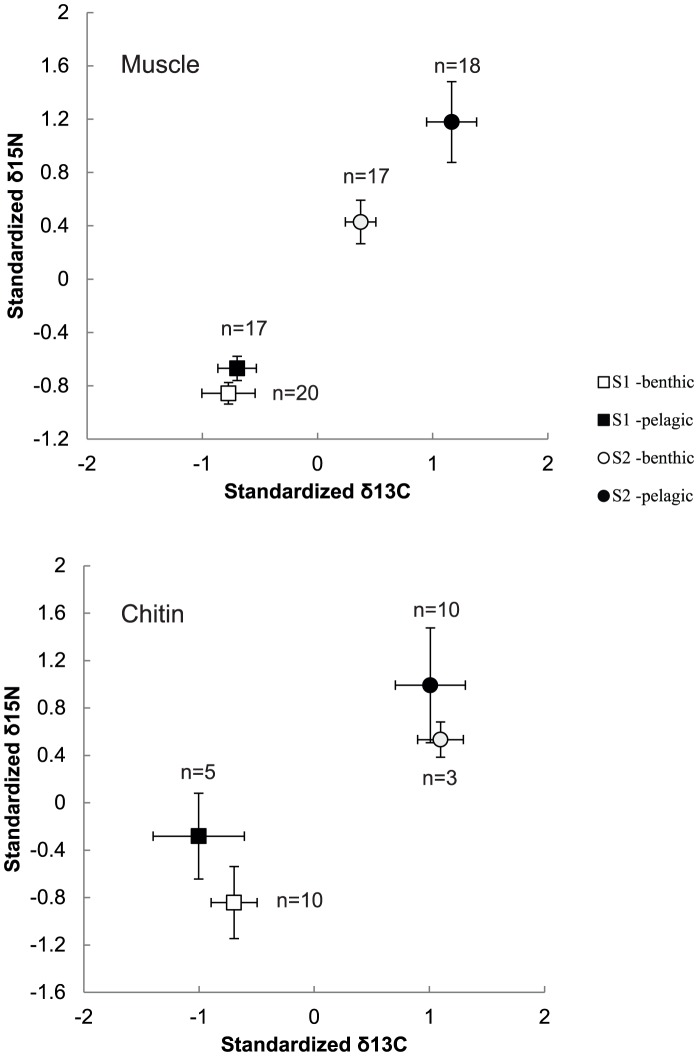
Standardized isotopic signatures of muscle tissue and chitin. Standardized isotopic signatures of δ^13^C and δ^15^N at stations S1 and S2 for muscle tissue and chitin showing relative differences between benthic and pelagic samples. Standardized values (*z*) were calculated as: [*z* = (*x*−*µ*)/*σ*] where x is the raw isotopic value, *µ* is the population mean (all raw isotopic values) and *σ* the standard deviation of the population. White markers denote benthic samples and black –pelagic samples. Whiskers show 95% confidence intervals.

The joint isotopic signal differed significantly between stations and between habitats (Table 2). Between the stations, muscle and chitin values were approximately 1‰ (δ^15^N) and 0.6‰ (δ^13^C) higher at station S2 (Table 1 and Fig. 2).

When the carbon and nitrogen isotope ratios were analyzed separately, we also found significant differences between the habitats, with adjusted mean δ^15^N being 0.14 and 0.25‰ lower in the benthic samples of muscle tissue and chitin, respectively (Table 2). The δ^13^C however, only differed significantly between habitats in muscle tissue at station S2 (ANCOVA with BL as covariate; *t*_2, 32_, = 2.7, *p* = 0.011), with adjusted mean δ^13^C being 0.15‰ higher in the pelagic zone. A tendency, albeit not significant, towards lower (muscle tissue) and higher (chitin) δ^13^C values in benthic compared to pelagic samples were observed (Table 1, Table 2).

In the pelagic habitats, correlations between δ^15^N and δ^13^C were moderate to high (*r* = 0.42; 0.69, station S1 and S2 respectively), whereas benthic correlations were considerably weaker (*r* = 0.17; 0.05, station S1 and S2 respectively).

### Biochemical indices for growth condition

There was a significant difference in protein:DNA ratio between the stations (*t*_1, 52_, = 2.6, *p* = 0.012) but not between the habitats (*t*_1, 52_, = −0.54, *p* = 0.59) and these results were not affected by BL (*t*_1, 52_, = −1.25, *p* = 0.22). The adjusted mean of log protein (log DNA as covariate; *t*_4, 53_, = 4.3, *p* = 0.0001) was slightly higher at station S2 indicating a higher growth status (*t*_4, 53_, = 2.7, *p* = 0.009).

C:N ratios differed significantly between the stations (*t*_1, 67_, = −2.6, *p* = 0.013) with slightly lower ratios at station S1, whereas no difference was found between the habitats (*t*_1, 67_, = 1.4, *p* = 0.16). C:N ratios were not affected by mysid size (*t*_1, 67_, = 0.9, *p* = 0.37).

### Population genetic structure

**Figure 3:**
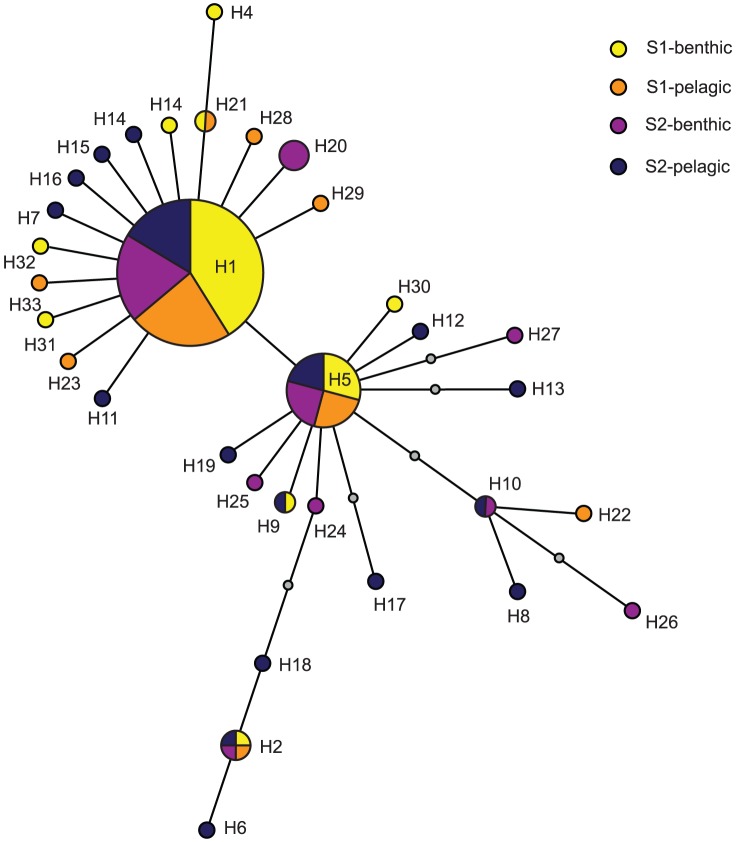
Minimum spanning tree depicting haplotypic relativeness.

A total of 33 mtDNA haplotypes were identified among the 160 individuals of *M. salemaai* (GenBank, accession numbers: JF279706-JF279873), six of which (H1, H2, H5, H8, H10, and H16) have been described previously [18], [69]. Diversity indices were higher at station S2 than S1 (Table 3) with a maximum of 18 haplotypes in the pelagic sample at station S2. At each station, the pelagic group displayed higher haplotype and nucleotide diversities than the benthic group, with benthic mysids at S1 having the lowest diversity (Table 3). Only 3 haplotypes were shared between the four sampled groups (H1, H2 and H5; Fig. 3), H1 being the most frequent. The remaining haplotypes occurred with a frequency ranging from 1 to 7 and many were unique to their respective sampling locations. The haplotypes did not cluster into any specific habitat or station and most of the unique or less frequent haplotypes were closely related to the dominating haplotypes (H1 and H5), differing by one or two mutational steps (Fig. 3).

**Table 3:** Molecular diversity indices.

Sample	*n*	Hn	Hd	π
S1-benthic	54	10	0.493	0.00167
S1-pelagic	35	9	0.620	0.00230
S2-benthic	35	9	0.706	0.00301
S2-pelagic	36	18	0.822	0.00429

Molecular diversity indices for benthic and pelagic samples at stations S1 and S2. *n* = sample size, Hn = number of haplotypes, Hd = haplotype diversity, π = nucleotide diversity.

Minimum spanning tree showing the relationship between haplotypes found in pelagic and benthic samples at station S1 and S2. The size of the circles is proportional to the relative frequency of haplotypes. The smallest colored circle represents one individual. Grey circles show missing or not sampled haplotypes.

Whereas no evidence for genetic differentiation was found based merely on haplotype frequency distributions (Fisher's exact test, *p*>0.09), the panmixia hypothesis was rejected based on low yet significant global genetic differentiation (Φ*_ST_* = 0.035 with station as grouping factor and Φ*_ST_* = 0.017 with habitat as grouping factor (AMOVA I and AMOVA II, Table 4) and pairwise comparisons (Table 5). Genetic variation between stations was significantly higher than between habitats, indicating that the genetic differentiation was attributable almost entirely to the geographic location of the sites and not to differences in migratory strategies. Moderate differentiation was observed between benthic groups (*F_ST_* = 0.053) as well as between the pelagic group at station S2 and the benthic group at station S1 (*F_ST_* = 0.054). All other pairwise comparisons were non-significant (Table 5), indicating a high degree of gene flow between these groups.

**Table 4:** Analysis of molecular variance table.

Source of variation	d.f.	SS		% variation	
Among groups	1	2.51*	0.69†	4.0*	−1.5†
Among populations within groups	2	0.97*	2.79†	−0.5*	3.2†
Within populations	156	95.9*	95.9†	97*	98†
Total		99.4*	99.4†		
Φ_ST_	0.035*	0.017†			
*P*	0.028*	0.030†			

Analysis of molecular variance table (AMOVA) based on four population samples (benthic and pelagic samples at stations S1 and S2). The samples are grouped either by station (*) or habitat (†).

**Table 5:** F_ST_ pairwise comparisons.

	S1-benthic	S1-pelagic	S2-benthic	S2-pelagic
S1-benthic		0.682	**0.0138**	**0.009**
S1-pelagic	−0.0094		0.207	0.173
S2-benthic	**0.0532**	0.0125		0.487
S2-pelagic	**0.0540**	0.0121	−0.0043	

Below diagonal: pairwise comparisons showing *F_ST_* values between benthic and pelagic mysids from station S1 and S2. Bold figures mark significance at the 0.05 level after correction with the modified FDR procedure [65]. Above diagonal: raw p-values.

## Discussion

### Habitat usage and resource partitioning

The stable isotope signatures in *M. salemaai* suggest that differences in resource partitioning occur between animals collected in the pelagia and close to the bottom. Higher δ^15^N- values in pelagic mysids suggest that they consume a higher proportion of isotopically heavy food (Table 2, Fig. 2) and it is likely that this isotopic signal results from a more carnivorous diet reasonably made up by zooplankton (cf. [70], [71]); which also was corroborated by δ^15^N-values of zooplankton sampled close to station S2 (7–9‰, 2007; [72]), i.e. approximately one trophic level below mysids in this study [41].

As expected, due to enrichment in ^15^N from incorporation of sewage-derived N from the STP effluent, and in ^13^C from the freshwater inflow and runoff to the bay, average isotopic signatures were higher at S2 than S1, and these differences were directly reflected in the δ^15^N-values of seston [0–10 m depth, 10 µm fraction (A. Zakrisson, Department of Systems Ecology, Stockholm University; personal communication)]. The summer average δ^15^N-signal of seston (June-September, 2008) was approximately 3 and 4.2‰ at sites that are in close proximity to stations S1 and S2 respectively, demonstrating that the δ^15^N discrepancy observed between mysids at stations S1 and S2 was due to station specific differences in baseline and not a result of different diets.

Between-habitat differences were consistent, with both pelagic groups having higher average values than respective benthic groups, both in muscle tissue and chitin. The small, yet significant, differences in δ^15^N values between the benthic and pelagic groups suggest consistent time-integrated differences in diet, both on the longer and shorter time scale. Muscle tissue signatures have a turnover of 2–3 months in mysids of this size [56] whilst chitin signatures represent diet consumed over the last few weeks. Thus, in combination, both tissues provide a rather conservative measure of the trophic position. Moreover, the diets of migrant and residents may partially overlap thus masking the differences in the diets. Indeed, both benthic and pelagic mysids may feed in the benthic habitat during daytime [13], and some zooplankton species distributed more or less evenly in the water column may be available for the residents as well as the migrants. The fact that δ^13^C at station S1 did not differ significantly between the habitats may indicate more a lack of difference in carbon isotopic composition of the ingested food between the habitats rather than similar diets. In omnivores, a true difference in diet can be hidden, because their isotopic signature often reflects that of their dietary protein. Thus, even a low intake of animal tissue in benthic mysids would produce relatively high imprints on the δ-values, suggesting a diet more similar to that of pelagic mysids than in reality. For example, omnivorous crayfish display trophic positions close to those of predatory fish –which is higher than would be expected from gut content analysis alone [73]. Mysids feeding on meiofauna, zooplankton resting eggs and small amphipods [74], [75] could have contributed to this apparent similarity in δ^13^C between the groups at station S1. The more carnivorous nature of pelagic mysids [13] is further supported by substantially stronger δ^15^N, δ^13^C-correlations as compared to benthic individuals that appear to have broader, more omnivorous diets. Polunin *et al.* [76] and Fanelli *et al.* [77] reported that stronger correlations usually were indicative of pelagic feeding organisms with a narrower diet composition compared to benthic feeders.

### Population structure and genetic differentiation

The genetic structure analysis suggests that significant genetic differentiation occurs between local *M. salemaai* populations, particularly between the bottom-collected animals. When accounting for the amount of molecular differentiation between the haplotypes (AMOVA), a significant fine-scale genetic structure was observed, with most haplotypes being unique to their respective sample locations. The most important observation is that in the continuous population of *M. salemaai*, bottom-dwelling mysids have a significant degree of genetic differentiation between the closely situated sites (∼20 km). Moreover, benthic mysids at station S1 had the lowest within-group diversity and were significantly different from not only the other benthic, but also the pelagic group at station S2. The differences observed are most likely related to the restricted migrations of mysids collected near the bottom at night compared to the pelagic individuals. If this is true, then pelagic individuals would contribute most to the gene flow in the population. Intuitively, this makes sense, since individuals performing extensive DVMs would be more susceptible to passive transportation by currents than non-migrating individuals.

Although many causes for the observed genetic differentiation within the mysid population inhabiting the area may exist, we suggest migratory behavior being an important factor affecting gene flow rate. In our study area, estuarine circulation is the main hydrodynamic mechanism accounting for the water exchange between Himmerfjärden Bay north of station S2 and the open coastal area where S1 is located [78], [79]. This means that there is a net influx of heavier sea water along the bottom and below the thermocline into the bay (main water exchange occurs through the strait marked with an arrow in Fig. 1), whereas freshwater runoff is transported out in the surface layer. Since adult mysids rarely migrate through the thermocline [4], [80], it is reasonable to hypothesize that mysids transported by currents would be carried with the mentioned bottom water flow into the bay, i.e. from the area where station S1 is situated into the area of station S2 (Fig. 1). Consequently, only mobile, pelagic S1-mysids would continuously be entering the S2 area via unidirectional current-transport, which could explain the significant genetic differentiation between pelagic S2 and benthic S1 mysids.

### Partial DVM as a form of partial migration

The combination of ecological and genetic markers, indicate that the *M. salemaai* population shows ecological niche partitioning, with two reasonably distinct ecotypes. The pelagic ecotype conducts a DVM, has higher trophic position, more uniform diet, and contributes largely to horizontal migrations and gene flow within a population, whereas the benthic ecotype is relatively sedentary, with limited (if any) DVM, is more omnivorous, presumably feeding to a larger extent on detritus, and has a spatially restricted gene flow. Undoubtedly, the patterns of mysid migration seen here strongly resemble those reported for partially migrating fishes, birds and amphibians. Although there are numerous studies demonstrating bimodal vertical zooplankton distributions [81], few have actually evaluated the consistency of a migratory or resident behavior (but see [82]).

As we found no evidence for significant genetic differentiation between benthic and pelagic mysids within stations, it is plausible that this trait is plastic as reported for several fish species [37]–[39]. When both feeding conditions [83] and predation pressures [84], [85] are variable over the season in a fairly predictable manner, it is reasonable to suggest that a flexible behavior would be more adaptive than a genetically fixed trait adapted to a specific set of conditions [86], [87]. Development and application of microsatellite markers would provide a better power and resolution than mtDNA for analyzing the genetic component in mysid migratory behavior [88]. However, although a genetic coupling to DVM has been observed in other zooplankters [82], [89], [90] on the basis of microsatellite markers [82], it is debatable whether a genetic basis for the observed behavioral differences would be advantageous for mysids. The observed genetic coupling to migration is ecologically relevant in daphnids [91], because these animals are parthenogenic, and have short generation times (days). By contrast, mysids are relatively long-lived (12–24 months in the Baltic Sea [19]) and therefore, their optimal genotypes should be able to respond effectively to seasonally variable cues (e.g. predation pressure, algal blooms, etc.) but not to cues on shorter time scales.

Moreover, as mysids are proposed to be food limited even when the zooplankton abundance is at its annual maximum [92], [93], it is also likely that the adoption of a migratory or resident strategy is driven by limited access to high quality food and competition for resources. When the zooplankton prey availability declines, the benefit of DVM will be reduced and a part of the population may become non-migratory. The non-migrating animals would stay permanently close to the bottom where the supply of low quality food, e.g. detritus, is stable and relatively high whereas predation pressure is low. Indeed, the benthic fish fauna in the study area is constituted mainly by small gobids (*Gobidae*) that primarily feed on less motile prey, such as benthic amphipods, harpacticoids and oligochaetes (unpublished data). Thus, the predation pressure from these fish is low compared to that of the much more abundant herring, which feed to a considerable extent on mysids [94].

If the partial DVM that we have observed is evolutionary stable and yields equal fitness benefits to both strategies [28], [32], we would expect that the condition of animals adopting these tactics would be equal or at least equalize after a certain time, given equal predation pressures. The fact that we did not find any between-habitat differences in either protein:DNA or C:N ratios that were used as proxies for protein and lipid accumulation, respectively, supports this idea and suggests that the cost/benefit ratio is similar for migrating and non-migrating individuals under given environmental conditions and mysid densities.

What determines an individual's decision to migrate or not is still debatable but seems to be linked to conditional cues, at least in fish [34], [38], [39]. Fish condition has been reported to be positively correlated with the probability of migration when costs of migration are prevalent but benefits include predation risk aversion [34]. Inversely, the condition can be negatively correlated to migration choice when the primary reason for migration is to relieve intraspecific competition [39]. Although we could not address this question directly in this study, it is clear that mysid growth rates and thus condition could be variable over the season. Lehtiniemi, Viitasalo and Kuosa [17] observed a cohort of *M. mixta* having a varying size distribution pattern during the growth season: a unimodal in late spring-early summer, a bi-modal in summer-early autumn, and a unimodal again in late autumn. This dynamics in individual body size indicates diverging growth patterns, with a part of the population growing considerably faster under a period of time. Although we did not observe differences in the growth proxies in the autumn, they may occur during other periods and under different environmental conditions. Assuming that such patterns are frequent, it is possible that growth rate could be the decisive factor regulating migration in mysids as found for partially migrating fish.

### Concluding remarks

We hypothesized that the apparent lack of migration in some mysids could have been attributed to either (i) interspecific differences in migration between the sibling species, (ii) an unsynchronized DVM within the homogenous population, i.e. non simultaneous migration towards the surface, or (iii) consistent, intraspecific divergent migratory tactics with distinct genetic morphs.

The first hypothesis could not be rigorously tested since too few *M. relicta* individuals were present in our samples. Persistent differences in the resource use and indications for restricted horizontal migration in the benthic part of the population were however found within the sampled *M. salemaai* population; demonstrating that these differences could not have arisen in a population conducting unsynchronized migrations (second hypothesis). Feeding on equal amounts of the same sources but at different times should have resulted in isotopic values more similar between benthic and pelagic mysids than those observed in our study. Finally, the lack of genetic differentiation at the level of mtDNA between the benthic and pelagic mysids suggests that the observed differences in migratory strategies are plastic. However, application of other genetic markers is needed to further investigate the genetic component of the divergent strategies and its interaction with the environment. In any case, our results provide evidence for the existence of two *M. salemaai* ecotypes that differ in their DVM behavior and feeding habits, without any clear differences in growth condition; thus resembling many cases of partial migration commonly observed in birds and fish populations.

Ignoring the existence of these ecotypes is likely to complicate e.g. food web mapping and modeling by means of stable isotopes as well as other environmental studies, such as monitoring of environmental contaminants [95]. Also, the limited capacity to horizontal migration in the benthic ecotype implies that recolonization after, for example, bottom hypoxia events may be hampered if this ecotype dominates locally. Studying environmental variability and feedbacks of food web dynamics on the presence and abundance of these ecotypes would help to understand ecological and evolutionary mechanisms of this diversification.
